# The efficacy and safety of colistimethate sodium in the treatment of carbapenem-resistant Gram-negative bacilli: a real-world observational study

**DOI:** 10.3389/fcimb.2026.1742142

**Published:** 2026-05-29

**Authors:** Tingyuan Zhang, Fan Zhang, Zhengrong Mao, Lei Li, Lihui Wang, Chao Qin, Dahuan Li, Jianwei Wang, Huaqiang Wang, Shaofang Wang, Huanzhang Shao

**Affiliations:** 1 Department of Critical Care Medicine, Henan Provincial People’s Hospital, Zhengzhou, Henan, China; 2 Department of Critical Care Medicine, People’s Hospital of Zhengzhou University, Zhengzhou, China; 3 Henan Key Laboratory for Critical Care Medicine, Zhengzhou, China; 4 Department of Critical Care Medicine, The First Affiliated Hospital of Henan University of Traditional Chinese Medicine, Zhengzhou, Henan, China; 5 Department of Respiratory Intensive Care Unit, Henan University Huaihe Hospital, Kaifeng, Henan, China; 6 Department of Critical Care Medicine, Henan Provincial Traditional Chinese Medicine Hospital, Zhengzhou, Henan, China; 7 Department of Critical Care Medicine, The Fifth Affiliated Hospital of Zhengzhou University, Zhengzhou, Henan, China; 8 Department of Emergency Intensive Care Unit, The First Affiliated Hospital of Henan University of Science and Technology, Luoyang, Henan, China; 9 Department of Critical Care Medicine, Puyang People’s Hospital, Puyang, Henan, China; 10 Department of Critical Care Medicine, Xuchang Central Hospital, Xuchang, Henan, China; 11 Department of Respiratory Intensive Care Unit, Anyang People’s Hospital, Anyang, Henan, China

**Keywords:** carbapenem-resistant Gram-negative bacilli, colistimethate sodium, efficacy, prospective, safety

## Abstract

**Introduction:**

As carbapenem-resistant Gram-negative bacilli (CR-GNB) has spread worldwide, colistimethate sodium (CMS) has been reintroduced into clinic, yet high-quality evidence remains scarce; therefore, this study—the first prospective, observational investigation in China—was designed to evaluate the clinical efficacy and safety of CMS in treating CR-GNB.

**Methods:**

Between November 2021 and November 2023, nine ICUs in Henan Province consecutively enrolled 222 patients aged ≥14 years with culture-confirmed CR-GNB infection who were planned to receive ≥7 days of intravenous CMS. The primary efficacy endpoints were clinical response and 30-day all-cause mortality; the main safety endpoint was CMS-associated acute kidney injury (AKI).

**Results:**

The overall clinical response rate was 82.5% (95% CI: 76.6%–87.3%), consisting of 17.1% complete resolution and 65.4% partial clinical improvement. The microbiological response rate was 79.0%, the bacterial clearance rate was 72.8%, and the 30-day all-cause mortality was 19.8%. Subgroup analyses showed that CR-GNB patients with single-site infection, isolated pulmonary infection, or monomicrobial infection achieved significantly better clinical efficacy. Multivariable regression analysis revealed that higher APACHE II scores (OR = 0.91), multi-site infection (OR = 0.26), and polymicrobial infection (OR = 0.35) were independent risk factors for poor clinical response. Meanwhile, multi-site infection (OR = 4.19) and polymicrobial infection (OR = 2.94) served as independent risk factors for 30-day all-cause mortality. In terms of safety, the overall incidence of adverse events (AEs) was 3.6%, and the incidence of acute kidney injury (AKI) was 20.4%, mostly classified as KDIGO stage I. In addition, patients with elevated baseline serum creatinine demonstrated a significantly higher risk of AKI.

**Discussion:**

Overall, CMS-based regimens achieved favorable efficacy in ICU patients with CR-GNB infections, with an acceptable overall safety profile.

## 1 Introduction

Carbapenem-resistant Gram-negative bacilli (CR-GNB) have spread globally, with increasing prevalence year by year. It is considered a formidable threat to public health worldwide. Moreover, the World Health Organization has listed them as “critical priority pathogens” requiring urgent attention (Mei et al., 2023; Run et al., 2025). More critically, CR-GNB infections are frequently underpinned by complex resistance mechanisms—including carbapenemase production, loss of outer-membrane porins, and overexpression of efflux pumps—that can act individually or synergistically to confer simultaneous resistance to multi class antibiotics (Bailong et al., 2025). Although several new β-lactam/β-lactamase inhibitor combinations have been approved for CR-GNB infections, they are not available in many countries and remain ineffective against some CR-GNB isolates, further constricting therapeutic options of CR-GNB (Jaffar A et al., 2025). Therefore, safe and effective therapeutic regimens are urgently needed to battle the escalating spread of CR-GNB.

Colistimethate sodium (CMS) is an inactive prodrug. It is hydrolyzed *in vivo* and yields the active colistin with antibacterial potency. CMS is routinely used in Europe for the treatment of bronchiectasis and chronic Pseudomonas aeruginosa infection (Charles S et al., 2024; Hongjiang et al., 2023). In recent years, CMS has been re-introduced into clinical use because of the worldwide dissemination of CR-GNB, the scarcity of novel antibiotics, and the continued misuse of antimicrobial agents (Evelina et al., 2018; Murat, 2016), making it one last-resort option for infections caused by carbapenem-resistant Pseudomonas aeruginosa (CRPA), carbapenem-resistant Klebsiella pneumoniae (CRKP), and carbapenem-resistant Acinetobacter baumannii (CRAB) (Cassandra, 2017). Many studies about its efficacy and safety have been published. For instance, a recent retrospective study reported that CMS achieved a clinical response rate of 62.96% in CR-GNB infected hematological patients with sepsis, with adverse events (AEs) documented in 19.75% of the cohort (Yan et al., 2025). Nevertheless, as an example of “old drug, new use,” the current safety and efficacy profile of CMS remains based predominantly on retrospective analyses. High-quality prospective evidence is urgently required to systematically evaluate the efficacy and safety of CMS for the treatment of CR-GNB.

This project is a prospective, multicenter, observational real-world study to explore the clinical efficacy and safety of CMS in the treatment of CR-GNB patients. As the first prospective study of CMS with CR-GNB in China, it provides evidence-based support for the use of CMS in the Chinese population.

## 2 Methods

### 2.1 Study population

This multicenter, observational study was conducted in the intensive care unit (ICU) of 9 hospitals in Henan Province, China, from November 1, 2021, to November 1, 2023. Before the first CR-GNB patient was enrolled at any site, the study protocol was approved by the local ethics committee of the participating centres. Thereafter, the investigation was conducted in strict accordance with the Declaration of Helsinki, the International Council for Harmonisation Good Clinical Practice (ICH-GCP) guidelines, and pertinent Chinese regulations. Written informed consent was obtained from every patient or, when the patient lacked decision-making capacity, from the legally authorised representative prior to any study-specific procedure. This study has been registered with the Chinese Clinical Trial Registry (ChiCTR2100054085, https://www.chictr.org.cn/showproj.html?proj=142825).

To ensure consistency among the nine centers and minimize assessment bias, all participating researchers received unified training before study started. The training covered the study protocol, definitions of endpoints, and evaluation criteria. Most importantly, standard operating procedures (SOPs) for clinical outcome assessment were established and uniformly implemented across all centers. Cross-center review meetings were called as needed to adjudicate complex cases. Therefore, uniform assessment criteria and internal quality control were implemented across all participating centers.

All enrolled CR-GNB patients were ≥ 14 years old with infection confirmed by positive culture and antimicrobial susceptibility testing of clinically relevant specimens and were treated with CMS—alone or in combination with other antibiotics. Concomitant antibiotics comprised a carbapenem, tigecycline, ceftazidime-avibactam, or another agent. The choice of concomitant antibiotics was based on testing results for antimicrobial susceptibility, clinical guidelines, infection severity, and at attending physician’s discretion. As guided by the pathogen’s susceptibility report and the International Consensus Guidelines for the Optimal Use of Polymyxins, CMS was initiated with an intravenous loading dose of 300 mg colistin base activity (CBA) (~9 million IU) infused over 0.5–1 h. The first maintenance dose was administered 12–24 h after the load. Patients with normal renal function received 300–360 mg CBA (~9-10.9 million IU) daily, divided into two 0.5–1 h infusions given every 12 h. For those with renal impairment, the CMS dose was adjusted according to guidelines in the **Supplementary Table 1**.

The inclusion criteria were: (1) ICU patients aged ≥ 14 years; (2) Documented CR-GNB infection; (3) Clinical decision that systemic antibiotic therapy including CMS was required, with an intended combined course of ≥ 7 days. The exclusion criteria were: (1) Age< 14 years; (2) Receipt of any polymyxin within 7 days before screening; (3) Known hypersensitivity to CMS; (4) Positive pregnancy test or current lactation; (5) Any other condition judged by the investigator to render enrollment inappropriate.

### 2.2 Clinical outcomes

The objective of this study was to evaluate the efficacy and safety of CMS in the treatment of CR-GNB infections. The primary efficacy endpoints were the clinical response to CMS therapy for CR-GNB and 30-day all-cause mortality, while the primary safety endpoint was the incidence of nephrotoxicity associated with CMS-containing regimens. Clinical response was defined as the attainment of either clinical improvement or clinical failure in CR-GNB-infected patients. Clinical improvement was defined as either complete resolution (disappearance of all infection-related signs and symptoms) or partial clinical improvement. Partial clinical improvement was defined as meeting at least two of the following criteria: resolution or reduction of fever (temperature ≤ 38.0 °C), improved respiratory status and oxygenation, decreased vasopressor support, improved general condition, and declining inflammatory markers (PCT, CRP), without the need for antimicrobial escalation and allowing safe discontinuation of CMS at the intended 14–21 days. Clinical failure was defined as persistent or worsening infection−related manifestations, requirement for antimicrobial therapy escalation, or infection−related death. Secondary efficacy endpoints included microbiological response (eradication vs. persistence of the CR-GNB isolate), composite success (simultaneous clinical and microbiological cure), bacterial clearance rate, length of ICU stay, and total hospitalization days.

### 2.3 Sample-size calculation

As a prospective, multicenter, observational study, the sample size was calculated on the basis of the primary endpoint, prior published data (Merlin et al., 2020; Yan et al., 2025), and consensus of multidisciplinary experts, resulting in a plan of enrolling 246 patients with CR-GNB infection. The calculation was performed as follows: the anticipated p (overall clinical improvement rate with CMS therapy for CR-GNB infection) was estimated at 70%. Using allowable error δ (the maximum non-significant difference between the sample rate and the true population rate) at 0.10, a two-sided α of 0.05, and a power (1 – β) of 90%, the required sample size was 221 patients. After accounting for a 10% dropout rate, a minimum of 246 patients had to be enrolled. The corresponding sample-size formula is:


$$N=p(1-p)\frac{{\left(\mu_{a}+\mu_{\beta }\right)}^{2}}{\delta^{2}}$$

where μ is the quantile of the standard normal distribution.

**Figure 1:**
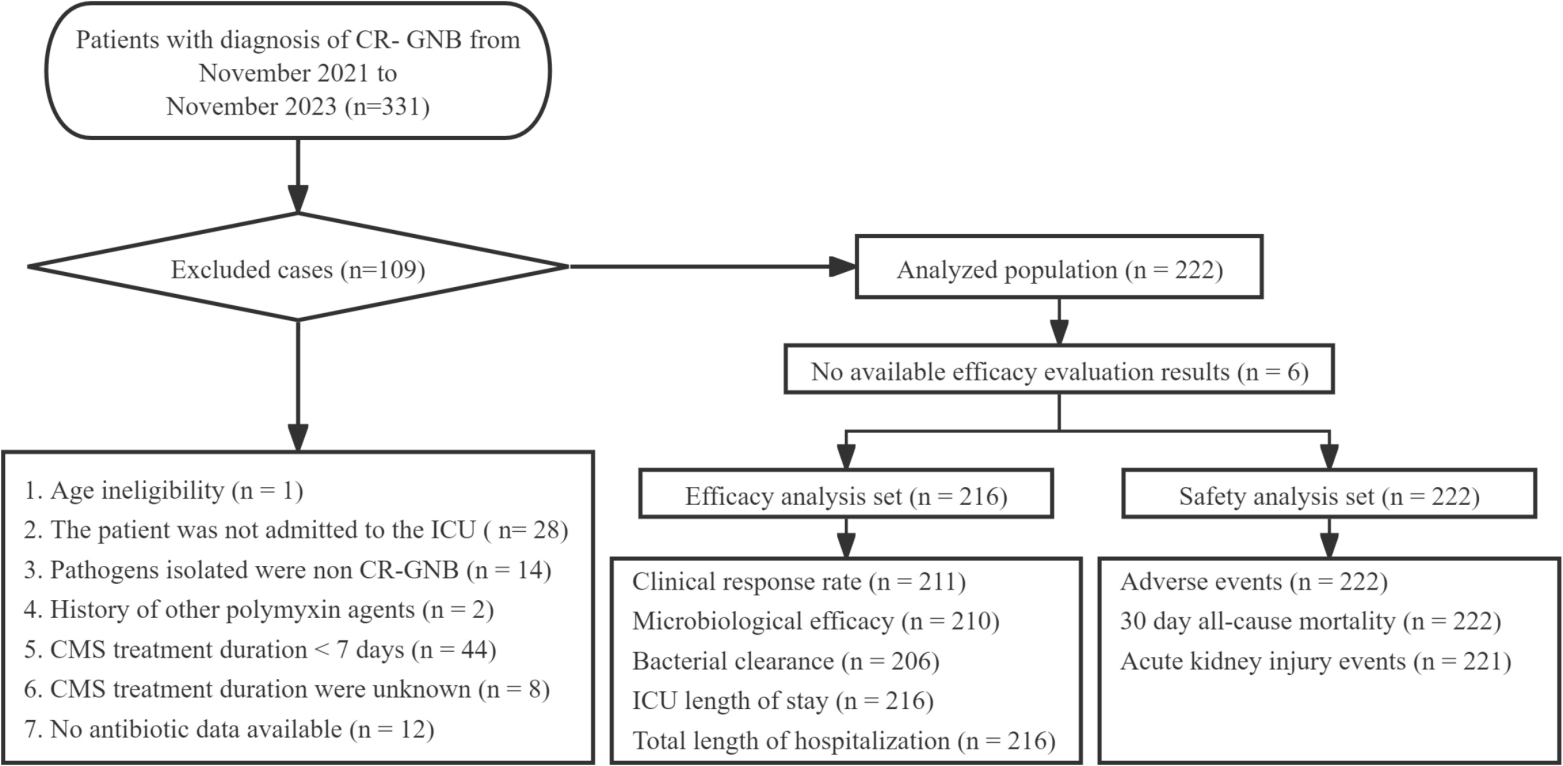
Patient flow diagram. Flow of patients diagnosed with carbapenem-resistant Gram-negative bacteria (CR-GNB) infection from November 2021 to November 2023. A total of 331 patients were initially enrolled; 109 were excluded, leaving 222 in the analyzed population and safety analysis set. After further exclusion of 6 patients with no evaluable efficacy data, 216 constituted the efficacy analysis set. Outcomes included clinical response, microbiological efficacy, bacterial clearance, length of stay, adverse events, 30-day all-cause mortality, and acute kidney injury. ICU intensive care unit, CMS Colistimethate sodium, AKI Acute kidney injury.

A total of 331 patients with CR-GNB infection were prospectively screened for this study. Among them, 109 patients were excluded due to ineligibility. The most common reason for exclusion was fewer than 7 days of CMS treatment, which consisted of 44 patients. The clinical characteristics of these excluded patients are presented in **Supplementary Table 2**. Finally, 222 patients with CR-GNB infection were included in the subsequent analyses. For efficacy analysis, 6 patients were excluded due to missing efficacy data. Accordingly, a total of 216 patients with CR-GNB infection constituted the efficacy analysis set. The patient-selection flowchart is presented in (**Figure 1**), including the reasons for exclusion, sample sizes of the efficacy-analysis set, the safety-analysis set, and the AKI-event analysis set.

### 2.4 Safety analysis

Throughout the study, CR-GNB patient safety was comprehensively evaluated by continuously recording all adverse events (AEs) and by applying the Kidney Disease Improving Global Outcomes (KDIGO) 2012 criteria to detect and grade any acute kidney injury (AKI), thereby providing an integrated assessment of CMS-associated toxicity.

### 2.5 Statistical analysis

Continuous variables are summarized as mean ± SD, median (IQR), and observed range (min–max). Categorical variables are reported as counts and percentages. Decimal places are rounded to one decimal for summary statistics, while the min–max values retain their original precision. The primary endpoint—clinical response rate—was compared between groups using the χ² test or Fisher’s exact test, as appropriate. Missing values of the SOFA and APACHE II scores were imputed using multiple imputation with predictive mean matching (PMM). A fixed random seed was set, and five imputed datasets were generated. Statistical results from the complete imputed datasets were subsequently pooled according to Rubin’s rules. To address potential collinearity among covariates, a stepwise logistic regression model was utilized to analyze the independent risk factors for both clinical response and 30-day all-cause mortality. Subgroup analyses of the primary endpoint are presented with 95% CIs calculated by the Clopper–Pearson method. For secondary endpoints, comparisons of variables between groups were conducted using the χ² test or Fisher’s exact test, as appropriate. Safety data are described as counts and percentages. All analyses were performed with SAS 9.4; and *p*-value< 0.05 was considered statistically significant.

## 3 Results

### 3.1 Baseline characteristics of CR-GNB patients receiving CMS therapy

Baseline characteristics of the 222 enrolled patients are summarized in **Table 1**. A total of 222 CR-GNB-infected patients were enrolled. We comprehensively summarize the baseline characteristics, including demographic characteristics, medical history, infection-related presentation and CMS administration details. The cohort was predominantly male (71.2%), with a median age of 64.0 years (IQR 53.0–73.0) and a median BMI of 23.7 kg/m² (IQR 21.0–26.0). Besides, the median SOFA score was 7.0 (IQR 5.0–9.0) and the median APACHE II score was 22.0 (IQR 17.0–26.0). Second, 121 (54.5%) patients had previously received antimicrobial therapy, while 172 (77.8%) patients had underlying diseases, among which cardiovascular diseases predominated, accounting for 54.3%. Third, most patients (96.4%) had pulmonary infection, accompanied by fever (68.0%) and dyspnea (15.3%). The predominant pathogen was carbapenem-resistant Acinetobacter baumannii (CRAB; 50.0%), followed by carbapenem-resistant Klebsiella pneumoniae (CRKP; 39.2%). Overall, 78.6% of CR-GNB strains were non-susceptible to at least six antimicrobial classes. Only 19 patients (8.6%) received CMS monotherapy; nearly half (45.5%) were treated with a combination of CMS plus a carbapenem, and 152 (68.5%) patients received CMS via intravenous infusion.

**Table 1:** Patient demographic information and baseline characteristics.

Variables	Values (*n*=222)
Gender, *n* (%)	
Male	158 (71.2)
Female	64 (28.8)
Age, years	
Mean ± SD	62.8 ± 15.2
Median (IQR)	64.0 (53.0, 73.0)
Min, Max	19, 94
≤ 65	117 (52.7)
> 65	105 (47.3)
BMI, kg/m² (*n*=138)	
Mean ± SD	23.9 ± 4.0
Median (IQR)	23.7 (21.0, 26.0)
Min, Max	13.5, 38.6
Missing	222 (84)
<18.5	14 (10.1)
18.5-24.9	70 (50.7)
25.0-30.0	45 (32.6)
≥30.0	9 (6.5)
SOFA score	
Mean ± SD	7.6 ± 3.4
Median (IQR)	7.0 (5.0, 9.0)
Min, Max	1, 25
Missing	222 (51)
APACHE II score	
Mean ± SD	21.8 ± 6.3
Median (IQR)	22.0 (17.0, 26.0)
Min, Max	7, 38
Missing	222 (40)
History of anti-infective drug use, *n* (%)	
Yes	121 (54.5)
No	101 (45.5)
Comorbid conditions, *n* (%)	
Yes	172 (77.8)
Cardiovascular disease	120 (54.3)
Diabetes mellitus	46 (20.8)
Chronic kidney disease	14 (6.3)
Chronic neurologic disease	42 (19.0)
Chronic liver disease	12 (5.4)
Chronic pulmonary disease	24 (10.9)
Malignant neoplasm	22 (10.0)
Peptic ulcer disease	6 (2.7)
Hematologic disease	2 (0.9)
Other	18 (8.1)
No	47 (21.3)
Missing	1 (0.45)
Sites of infection, *n* (%)	
Pulmonary infection only	161 (72.5)
Pulmonary + other sites	53 (23.9)
Pulmonary + abdominal/biliary	5 (2.3)
Pulmonary + abdominal/biliary + bloodstream	8 (3.6)
Pulmonary + intra-cranial	6 (2.7)
Pulmonary + intra-cranial + CNS	1 (0.5)
Pulmonary + urinary tract	5 (2.3)
Pulmonary + skin/soft tissue	2 (0.9)
Pulmonary + bloodstream	16 (7.2)
Pulmonary + bloodstream + urinary tract	1 (0.5)
Pulmonary + bloodstream + CNS	2 (0.9)
Pulmonary + CNS	7 (3.2)
Abdominal/biliary only	3 (1.4)
Abdominal/biliary + other (intra-ocular)	1 (0.5)
Appendicitis + abdominal/biliary	1 (0.5)
Bloodstream + urinary tract	1 (0.5)
Intra-cranial infection only	2 (0.9)
Pathogen profile, *n* (%)	
CRAB only	84 (37.8)
CRAB + other pathogens	27 (12.2)
CREC	5 (2.3)
CRKP only	43 (19.4)
CRKP + other pathogens	44 (19.8)
CRPA only	16 (7.2)
CRPA + other pathogens	3 (1.4)
Bacterial load, CFU/mL, *n* (%)	
10³	5 (13.5)
10^4^	14 (37.8)
10^5^	16 (43.2)
10^6^	2 (5.4)
Missing	222 (185)
No. of MDR-GNB isolates	
1	52 (61.9)
2	20 (23.8)
3	7 (8.3)
4	5 (6.0)
Missing	222 (138)
No. of resistant antibiotic classes, *n* (%)	
1	1 (0.8)
2	2 (1.5)
3	7 (5.3)
4	7 (5.3)
5	11 (8.4)
6	103 (78.6)
Missing	222 (91)
Mean ± SD	5.5 ± 1.0
Median (IQR)	6.0 (6.0, 6.0)
Min, Max	1, 6
Infection-associated symptoms, *n* (%)	
Fever	151 (68.0)
Dyspnea/respiratory failure	34 (15.3)
Productive cough	23 (10.4)
Altered consciousness/loss of consciousness	6 (2.7)
Chest tightness/shortness of breath	13 (5.9)
Others	20 (9.0)
Treatment regimen, *n* (%)	
CMS + carbapenem	101 (45.5)
CMS + tigecycline	44 (19.8)
CMS + carbapenem + tigecycline	18 (8.1)
CMS + fosfomycin	1 (4.5)
CMS + other (guideline-recommended)	39 (17.6)
CMS monotherapy	19 (8.6)
Duration of CMS therapy, days	
Mean ± SD	13.0 ± 6.5
Median(IQR)	11.0 (9.0, 15.0)
Min, Max	7, 49
Route of CMS administration, *n* (%)	
Intravenous infusion	101 (45.5)
Nebulization	70 (31.5)
Nebulization + intravenous infusion	51 (23.0)
Dose adjustment, *n* (%)	
Yes^*^	2 (0.9)
No	200 (99.1)
Temporary discontinuation, *n* (%)	
Yes^‡^	1 (0.5)
No	221 (99.5)

IQR interquartile range, BMI body mass index, CNS central nervous system,CRAB carbapenem-resistant Acinetobacter baumannii, CREC carbapenem-resistant Escherichia coli, CRKP carbapenem-resistant Klebsiella pneumoniae, CRPA carbapenem-resistant Pseudomonas aeruginosa, No. Number, MDR-GNB multidrug-resistant Gram-negative bacilli, ICU intensive care unit, CMS colistimethate sodium.

* loading dose (n=1), route switch (n=1).

‡ Hospital discharge (n=1).

### 3.2 Clinical outcomes and factors

Among the 222 enrolled patients, 216 had at least one post-baseline efficacy assessment and constituted the clinical outcome analysis set. Five of these lacked a definitive primary-endpoint evaluation, leaving 211 patients for the main clinical response and subgroup analyses. Overall, 174 patients (82.5%, 95% CI 76.6%-87.3%) achieved clinical effectiveness: 36 (17.1%) were classified as complete resolution and 138 (65.4%) as partial improvement; 37 patients (17.5%) were judged clinical failures (**Table 2**). Subgroup analyses showed that clinical effectiveness was significantly higher in patients with single-site infection than in those with multi-site infection (88.4% vs 66.1%, *p*-value< 0.001). Likewise, clinical effectiveness were superior for pulmonary infection only versus pulmonary plus extrapulmonary infection (88.2% vs 64.0%, *p*-value< 0.001) and for monomicrobial versus polymicrobial infection (86.3% vs 75.0%, *p*-value = 0.040) (**Table 2**). Furthermore, univariate analyses identified that higher APACHE II scores (OR 0.90, 95% CI 0.85-0.96, *p*-value = 0.001), multi-site infection (OR 0.26, 95% CI 0.12–0.54, *p*-value = 0.0003), and polymicrobial infection (OR 0.48, 95% CI 0.23–0.98, *p*-value = 0.043) were risk factors for reduced clinical effectiveness. In multivariable analysis, age, SOFA score, APACHE II score, infection site and pathogenic pathogen were included in the model. Among these, APACHE II score (OR 0.91, 95% CI 0.84-0.97, *p*-value = 0.009), multiple sites (OR 0.26, 95% CI 0.12-0.58, *p*-value = 0.001), and polymicrobial infection (OR 0.35, 95% CI 0.15-0.79, p-value = 0.01) were independent risk factors for poor clinical efficacy (**Table 3**).

**Table 2:** Clinical response in the overall population and subgroup analyses.

Variables	*n*/*N^a^*	Clinical response (%), 95% CI	*p*-value
Clinical efficacy	174/211	82.5, 76.6-87.3	—
Complete resolution	36	17.1	
Partial improvement	138	65.4	
Clinical failure	37	17.5	
Subgroup clinical response			
Age			0.187
≤ 65	96/112	85.7, 77.8-91.6	
> 65	78/99	78.8, 69.4-86.4	
Gender			0.834
Male	124/151	82.1, 75.1-87.9	
Female	50/60	83.3, 71.5-91.7	
BMI (*n*=130)			0.248
<18.9	13/14	92.9, 66.1-99.8	
18.9-25.0	57/67	85.1, 74.3-92.6	
25-30	32/41	78.0, 62.4-89.4	
≥30	5/8	62.5, 24.5-91.5	
Sites of infection			<0.001^b^
Single site	137/155	88.4, 82.2-93.0	
Multiple sites	37/56	66.1, 52.2-78.2	
Pulmonary involvement			<0.001^b^
Pulmonary only	135/153	88.2, 82.0-92.9	
Pulmonary + other sites	32/50	64.0, 49.2-77.1	
Non-pulmonary	7/8	87.5, 47.3-99.7	
Pathogen			0.040^b^
Monomicrobial	120/139	86.3, 79.5-91.6	
CRAB	65/79	82.3, 72.1-89.9	
CRKP	37/43	86.0, 72.1-94.7	
CREC	5/5	100.0	
CRPA	13/14	92.9, 66.1-99.8	
Polymicrobial	54/72	75.0, 63.4-84.5	
Comorbidities			0.682
Yes	135/165	81.8, 75.1-87.4	
No	38/45	84.4, 70.5-93.5	
Prior antibiotic use			0.205
Yes	93/117	79.5, 71.0-86.4	
No	81/94	86.2, 77.5-92.4	
Treatment duration, days			0.871
≤ 11	97/117	82.9, 74.8-89.2	
> 11	77/94	81.9, 72.6-89.1	
Route of CMS administration			0.323
Intravenous infusion	79/100	79.0, 69.7-86.5	
Nebulization + intravenous infusion	42/51	82.4, 69.1-91.6	
Nebulization	53/60	88.3, 77.4-95.2	
Treatment regimen			0.414
CMS monotherapy	15/16	93.8, 69.8-99.8	
CMS + carbapenem	80/99	80.8, 71.7-88.0	
CMS + tigecycline	33/42	78.6, 63.2-89.7	
CMS + carbapenem + tigecycline	12/16	75.0, 47.6-92.7	
CMS + other	34/38	89.5, 75.2-97.1	

*n:* The number of clinically effective patients. *N:* the total number of patients in subgroups.

a: Five of these lacked a definitive primary-endpoint evaluation, leaving 211 patients for the main clinical response and subgroup analyses. b: Bold p-values indicate statistically significant differences (*p* < 0.05). Significant associations were found for sites of infection (*p* < 0.001), pulmonary involvement (*p* <0.001), and pathogen type (*p* = 0.040).

**Table 3:** The results of univariable and multivariable analyses of factors associated with clinical response.

Variables	Events/Samples	Univariable		Multivariable^a^	
		OR (95% CI)	*p*	OR (95% CI)	*p*
Age			0.189		0.147
≤ 65	96/112	Ref.		Ref.	
> 65	78/99	0.619 (0.303-1.266)		0.551 (0.242-1.225)	
Gender			0.834		
Male	124/151	Ref.			
Female	50/60	1.089 (0.491-2.414)			
SOFA score^b^	174/211	0.931 (0.850-1.022)	0.125	0.910 (0.811-1.016)	0.097
APACHE II score^b^	174/211	0.902 (0.845-0.958)	0.001^c^	0.905 (0.837,0.972)	0.009^c^
Sites of infection			0.0003^c^		0.001^c^
Single site	137/155	Ref.		Ref.	
Multiple sites	37/56	0.256 (0.122, 0.536)		0.261 (0.116,0.581)	
Pathogen			0.043^c^		0.012^c^
Monomicrobial	120/139	Ref.		Ref.	
Polymicrobial	54/72	0.475 (0.231-0.976)		0.351 (0.153, 0.788)	
Comorbidities^b^			0.694		
No	38/45	Ref.			
Yes	136/166	0.835 (0.317-1.957)			
Prior antibiotic use			0.207		
No	81/94	Ref.			
Yes	93/117	0.622 (0.297-1.301)			
Treatment duration, days			0.981		
≤ 11	97/117	Ref.			
> 11	77/94	0.999 (0.947-1.055)			
Route of CMS					
IV	79/100	Ref.			
Neb. + IV	42/51	1.241 (0.522-2.949)	0.626		
Neb.	53/60	2.013 (0.799-5.068)	0.138		
Treatment regimen			0.414		
CMS monotherapy	15/16	Ref.			
CMS + CBP	80/99	0.281 (0.035-2.107)	0.232		
CMS + TGC	33/42	0.244 (0.028-2.107)	0.199		
CMS + CBP + TGC	12/16	0.200 (0.020-2.033)	0.174		
CMS + other	34/38	0.567 (0.058-5.507)	0.624		

OR odds ratio, Ref. reference, IV intravenous infusion, Neb. Nebulization, CBP carbapenem, TGC tigecycline, *p p*-value.

a Given multicollinearity among variables, forward and backward stepwise regression was performed for multivariate analysis. The variables retained in the stepwise regression model were age, SOFA score, APACHE II score, sites of infection and pathogens. Variance inflation factor (VIF) was used to detect multicollinearity of each variable, with the VIF values of 1.055, 1.058, 1.102, 1.026 and 1.067 respectively, indicating weak multicollinearity.

b Multiple imputation was adopted to handle missing data, with comorbidities, SOFA score and APACHE II score imputed. Univariate and multivariate analyses were subsequently performed based on the imputed datasets. c: Bold p-values indicate statistical significance (*p* < 0.05). Significant associations with clinical response were found for APACHE II score (*p* = 0.001 in univariable; *p* = 0.009 in multivariable), sites of infection (*p* = 0.0003 in univariable; *p* = 0.001 in multivariable), and pathogen type (*p* = 0.043 in univariable; *p* = 0.012 in multivariable).

The microbiological efficacy rate was 79.0% (95% CI 72.9-84.3), the overall clinical efficacy rate was 78.2% (95% CI 72.0-83.6), and the bacterial clearance rate was 72.8% (95% CI 66.2-78.8). Besides, the median duration of mechanical ventilation was 14.0 days (IQR 9.0–20.0), the median ICU length of stay was 21.0 days (IQR 15.0–28.0), and the median total hospitalization length was 26.0 days (IQR 19.0–37.0) (**Supplementary Table S3**). The 30-day all-cause mortality, attributable to multiple causes as detailed in **Supplementary Table S4**, was 19.8%; among the deceased patients, ineffective anti-infective treatment accounted for 15 deaths (50%), which represented the primary cause. The risk factors for 30-day all-cause mortality identified by univariate analyses included multi-site infection (OR 2.86, 95% CI 1.30-6.31, p-value = 0.009) and polymicrobial infection (OR 2.25, 95% CI 1.04-4.91, p-value = 0.04) which were associated with increased odds of death (**Table 4**). Multivariable analysis showed that multiple-site infection (OR 4.19, 95% CI 1.70-10.73, p-value = 0.002) and polymicrobial infection (OR 2.94, 95% CI 1.25-7.089, p-value = 0.01) were independent risk factors for CR-GNB infection, whereas treatment duration > 11 days of CMS (OR 0.34, 95% CI 0.13-0.82, p-value = 0.02) was an independent protective factor (**Table 4**).

**Table 4:** The results of univariate and multivariate analyses of 30 day all-cause mortality.

Variables	Events/Samples	Univariable		Multivariable^a^	
		OR (95% CI)	*p*	OR (95% CI)	*p*
Age			0.138		0.061
≤ 65	12/117	Ref.		Ref.	
> 65	18/105	1.810 (0.827-3.964)		2.357 (0.981-5.992)	
Gender			0.063		0.052
Male	17/158	Ref.		Ref.	
Female	13/64	2.114 (0.930-4.658)		2.367 (0.984-5.691)	
SOFA score^b^		1.110 (0.993-1.239)	0.061		
APACHE II score^b^		1.036 (0.975-1.103)	0.261		
Sites of infection			0.009^c^		0.002^c^
Single site	16/163	Ref.		Ref.	
Multiple sites	14/59	2.858 (1.296-6.306)		4.190 (1.702-10.727)	
Pathogen			0.041^c^		0.014^c^
Monomicrobial	15/148	Ref.		Ref.	
Polymicrobial	15/74	2.254 (1.035-4.911)		2.944 (1.249-7.089)	
Comorbidities^b^			0.053		0.061
No	2/47	Ref.		Ref.	
Yes	28/175	4.286 (1.222-27.185)		4.316 (1.150-28.460)	
Prior antibiotic use			0.072		
No	9/101	Ref.			
Yes	21/121	2.147 (0.935-4.926)			
Treatment duration, days			0.129		0.021^c^
≤ 11	21/121	Ref.		Ref.	
> 11	9/101	0.9369 (0.858-1.020)		0.339 (0.128-0.821)	
Route of CMS					
IV	12/101	Ref.			
Neb. + IV	7/51	1.180 (0.434-3.206)	0.746		
Neb.	11/70	1.383 (0.573-3.340)	0.471		
Treatment regimen					
CMS monotherapy	1/19	Ref.			
CMS + CBP	20/101	4.441 (0.559-35.249)	0.158		
CMS + TGC	3/44	1.316 (0.128-13.519)	0.817		
CMS + CBP + TGC	3/18	3.597 (0.338-38.250)	0.289		
CMS + other	3/40	1.458 (0.142-15.012)	0.751		

OR odds ratio, Ref. reference, IV intravenous infusion, Neb. Nebulization, CBP carbapenem, TGC tigecycline, *p p*-value.

a Given multicollinearity among variables, forward and backward stepwise regression was performed for multivariate analysis. The variables retained in the stepwise regression model included age, gender, sites of infection, pathogen, comorbidities and treatment duration. Variance inflation factor (VIF) was utilized to examine multicollinearity of the above variables, with corresponding VIF values of 1.049, 1.132, 1.166, 1.062, 1.007 and 1.078, respectively, indicating weak multicollinearity.

b Multiple imputation was adopted to handle missing data, with comorbidities, SOFA score and APACHE II score imputed. Univariate and multivariate analyses were subsequently performed based on the imputed datasets. c: Bold p-values indicate statistical significance (*p* < 0.05). Significant associations with 30-day all-cause mortality were found for sites of infection (*p* = 0.009 in univariable; *p* = 0.002 in multivariable), pathogen type (*p* = 0.041 in univariable; *p* = 0.014 in multivariable), and treatment duration (*p* = 0.021 in multivariable).

### 3.3 Safety

Among the 222 patients receiving CMS, 8 (3.6%) patients experienced at least one AE, 6 had any AE, whereas 2 developed AEs that required treatment interruption: one for hepatic impairment and the other for renal impairment (**Supplementary Table S5**). Subsequently, for the assessment of AKI, one patient with missing data was excluded, leaving 221 patients evaluable for AKI analysis. Overall, 45 of 221 patients (20.4%) developed AKI. The majority (42.2%) were classified as KDIGO stage I. There were 17 patients (7.7% of the 221) experienced prolonged hospitalization due to an episode of AKI. Moreover, among the 38 patients with High baseline SCr, the incidence of AKI was markedly higher at 52.6%, and half of these cases (50%) were KDIGO stage 1. In contrast, patients with normal or low baseline SCr experienced AKI less frequently, at rates of 26.3% and 8.6%, respectively (**Supplementary Table S6**).

## 4 Discussion

CMS, regarded as a “last-resort” agent against CR-GNB infections, is now used worldwide (Mohamed Abd El-Gawad et al., 2020; Ulrike et al., 2021). Nevertheless, the utilization of CMS is confronted with multiple challenges, including pronounced nephrotoxicity, a narrow therapeutic window, and variable efficacy across different regions and populations (Aftab Hossain et al., 2024; Ji-Young et al., 2024; Xiaolan et al., 2024). In recent years, the escalating CR-GNB infections has driven increasing use of CMS, especially in low- and middle-income countries where access to novel antibacterial agents remains limited (Rachel et al., 2025; Yatin et al., 2022). In China, CMS use has also been rising; however, robust clinical data remain scarce, particularly lack of data from multicentre, prospective, real-world studies. As one first prospective, multicenter study in China, this investigational study aims to fill that gap by offering Chinese experience and more robust real-world clinical evidence on CMS therapy for patients with CR-GNB infections.

This study prospectively evaluated the efficacy and safety of CMS for the treatment of CR-GNB infections in the ICU population. Overall, CMS-based regimens achieved a clinical response rate of 82.5%, with 30-day all-cause mortality of 19.8% and AKI documented in 20.4% of patients, the majority classified as KDIGO stage 1. The overall prognosis of this study was better compared to that of multiple previous studies (Di et al., 2024). Compared with the 46.5% 30-day mortality rate of patients with CR-GNB reported in the ALARICO study, the mortality rate in this study was significantly reduced (Giusy et al., 2024). Further comparative analysis suggested that heterogeneity of results across different studies were likely attributable the differences in the patients baseline characteristics. In the ALARICO study, 39.6% of the enrolled CR-GNB patients developed septic shock and had more types of underlying diseases. In addition, in our study, CRAB accounted for 50% of the pathogens; this species has higher susceptibility to colistin than other CR-GNB strains, enabling CMS to exert greater bactericidal activity (Siqin et al., 2024), whereas the ALARICO study mainly reported KPC-producing Klebsiella pneumoniae. Although the different baseline characteristics indicated different patient population, the results of our study confirmed that CMS treatment could indeed benefit patients with CR-GNB to a certain extent. Another point worthy of attention is that the CMS regimen and administration route may also be key factors affecting the prognosis of CR-GNB. A previous study reported that the overall clinical response rate of CR-GNB patients receiving nebulized CMS was 79.3% (Yao et al., 2026), which was different from the 88.3% clinical response rate of nebulized CMS in our study. This therapeutic heterogeneity may also be part of the reason for the discrepancies in results from different studies. Therefore, different administration regimens, combined medication regimens, and routes of CMS are also important directions for the treatment of patients with CR-GNB to be considered.

Subgroup analyses revealed that patients with single-site infection (88.4% vs 66.1%), isolated pulmonary infection (88.2% vs 64.0%), or monomicrobial infection (86.3% vs 75.0%) achieved significantly higher clinical response rates than their respective complicated counterparts. Multivariable analysis further confirmed that multiple-site infection (OR = 0.26), polymicrobial pathogen (OR = 0.35), and a high APACHE II score (OR = 0.91) were independent risk factors for clinical failure, which is consistent with the retrospective cohort reported by Ping Yang et al (Ping et al., 2025). These findings indicate that the anatomic extent of infection, rather than the specific pathogen species, is likely the principal determinant of CMS treatment outcome; consequently, clinical management should prioritize containment of the infectious focus over mere escalation of antimicrobial therapy.

The 30-day mortality in the overall cohort of this study was 19.8%, which is highly consistent with the findings from the INCREMENT cohort (20%) and a meta-analysis report (22.5%–24.5%) (Wei et al., 2024; Zaira Raquel et al., 2017), reaffirming the survival benefit of CMS-based regimens in real-world practice. Importantly, unlike previous studies that identified the SOFA or APACHE II score as independent prognostic factors (Danyang et al., 2023; Fan et al., 2025; Min et al., 2022), multivariable analysis in the present study revealed that multiple-site infection (OR = 4.19) and polymicrobial pathogen (OR = 2.94) were independent predictors of 30-day mortality, indicating that the anatomical extent of infection exerts a more immediate influence on short-term survival than global physiological severity scores.

In this study, a total of 8 patients with CR-GNB infections experienced at least one AE during CMS treatment, among whom 2 discontinued CMS therapy due to renal or hepatic toxicity. Compared with similar studies, the incidence of AEs in the present study was relatively lower (Di et al., 2024; Jiale et al., 2023; Xiaojuan et al., 2021). Although baseline characteristics of patients enrolled in different studies were not comprehensively compared, the results still indicate to a certain extent that CMS may be favorable tolerability in the treatment of CR-GNB infections. For CMS-associated nephrotoxicity, the incidence of CMS-related AKI in this study was 20.4%, which was lower than the previously reported AKI incidences of 40.0% and 46.3% for CMS and colistin respectively (Pınar et al., 2024; Rima et al., 2023), and comparable to those of polymyxin B (28.63%) and ceftazidime/avibactam therapy (7/31) (Xiao-Li et al., 2022; Zihao et al., 2023). More importantly, among the 38 patients with high baseline SCr, the incidence of AKI was significantly higher at 52.6%, with half (50%) classified as KDIGO stage 1. In contrast, AKI occurred in 26.3% of those with normal baseline Scr and in only 8.6% of those with low baseline Scr. These findings indicate that patients with pre-existing renal impairment are at greater risk of AKI, and renal function monitoring should be emphasized during CMS administration in such individuals.

As a prospective, multicenter, non-interventional, observational study, this study cannot directly compare the efficacy of CMS with that of β-lactam/β-lactamase inhibitors because of the absence of randomization. In addition, residual confounding inherent to the observational design (e.g., severity of underlying diseases, timing of combination antibiotics, infection-control capacity across centers) cannot be fully eliminated statistically. A specific limitation worth emphasizing is the lack of critical detailed data and in-depth analysis regarding CMS-associated AKI. Besides, the 30-day follow-up precludes assessment of long-term renal function and neurotoxicity, while routine therapeutic drug monitoring is lacking, leaving drug exposure undefined. Finally, the pathogen distribution was dominated by CRAB and CRKP, with a small CRPA sample, so extrapolation demands caution. We will continue to refine our conclusions by extending follow-up, adding a therapeutic drug monitoring sub-cohort, and collecting more detailed data on CMS-associated AKI in future studies.

## 5 Conclusions

In this prospective, multicentre, real-world study of Chinese ICU patients with CR-GNB, CMS-based treatment achieved an 82.5% clinical response and 72.8% microbiological eradication. These results showed the potential of CMS-containing regimens for treating CR-GNB infection in critically ill ICU patients, with an overall acceptable safety profile and a low incidence of adverse events. In addition, baseline renal function may affect the occurrence of CMS-associated acute kidney injury, supporting routine renal function monitoring throughout the treatment course.

## Data Availability

The raw data supporting the conclusions of this article will be made available by the authors, without undue reservation.
