# Supplementary material for: The efficacy and safety of colistimethate sodium in the treatment of carbapenem-resistant Gram-negative bacilli: a real-world observational study
Source: Front Cell Infect Microbiol. 2026 May 29;16:1742142. doi: 10.3389/fcimb.2026.1742142 (PMC13259746; doi:10.3389/fcimb.2026.1742142)
Supplement: Supplementary file 1 [file Table1.docx]

**Supplementary Table S1** CMS Daily Dosage Form

| Creatinine Clearance (mL/min) | Achieve a Mean Steady-State Plasma Colistin Concentration (Css,avg) of 2 mg/L | |
| --- | --- | --- |
|  | mg CBA/d | 1 million IU/d |
| 0 | 130 | 3.95 |
| 5-＜10 | 145 | 4.40 |
| 10-＜20 | 160 | 4.85 |
| 20-＜30 | 175 | 5.30 |
| 30-＜40 | 195 | 5.90 |
| 40-＜50 | 220 | 6.65 |
| 50-＜60 | 245 | 7.40 |
| 60-＜70 | 275 | 8.35 |
| 70-＜80 | 300 | 9.00 |
| 80-＜90 | 340 | 10.30 |
| ≥90 | 360 | 10.90 |

CBA colistin base activity, CMS colistimethate sodium, Css,avg average steady-state plasma concentration.

The daily dose is divided into 2 administrations given every 12 h.
