# Supplementary material for: The efficacy and safety of colistimethate sodium in the treatment of carbapenem-resistant Gram-negative bacilli: a real-world observational study
Source: Front Cell Infect Microbiol. 2026 May 29;16:1742142. doi: 10.3389/fcimb.2026.1742142 (PMC13259746; doi:10.3389/fcimb.2026.1742142)
Supplement: Supplementary file 2 [file Table2.docx]

**Supplementary Table S2** Basic characteristics of patients with CMS duration less than 7 days

| **Variables** | **Values (*n*=44)** |
| --- | --- |
| Gender, *n* (%) |  |
| Male | 30 (68.2) |
| Female | 14 (31.8) |
| Age, years |  |
| Mean ± SD | 60.04 ± 14.25 |
| Median (IQR) | 60.0 (50.0, 71.0) |
| Min, Max | 24, 88 |
| Missing | 1 |
| SOFA score |  |
| Mean ± SD | 7.9 ± 2.9 |
| Median (IQR) | 8.0 (5.0,10.0) |
| Min, Max | 2, 13 |
| Missing | 13 |
| APACHE II score |  |
| Mean ± SD | 22.4 ± 6.9 |
| Median (IQR) | 23.5 (17.5, 26.5) |
| Min, Max | 4, 35 |
| Missing | 8 |
| Sites of infection, *n* (%) |  |
| Single site | 32 (72.7) |
| Multiple sites | 12 (27.3) |
| Pathogen |  |
| Monomicrobial | 31 (70.5) |
| Polymicrobial | 13 (29.5) |
| Pathogen profile, *n* (%) |  |
| CRAB only | 23 (52.3) |
| CRKP only | 5 (13.4) |
| CRAB + other pathogens | 7 (15.9) |
| CRKP + CRAB | 5 (13.4) |
| CRKP + other pathogens | 1 (2.3) |
| CRPA only | 3 (6.8) |
| Comorbid conditions, *n* (%) |  |
| No | 11 (25.0) |
| Yes | 33 (75.0) |

IQR interquartile range, CRAB carbapenem-resistant *Acinetobacter baumannii*, CRKP carbapenem-resistant *Klebsiella pneumoniae*, CRPA carbapenem-resistant *Pseudomonas aeruginosa*
