# Supplementary material for: The efficacy and safety of colistimethate sodium in the treatment of carbapenem-resistant Gram-negative bacilli: a real-world observational study
Source: Front Cell Infect Microbiol. 2026 May 29;16:1742142. doi: 10.3389/fcimb.2026.1742142 (PMC13259746; doi:10.3389/fcimb.2026.1742142)
Supplement: Supplementary file 3 [file Table3.docx]

**Supplementary Table S3** The analysis of secondary endpoints

| **Variables** | **Clinical response, values (*n*=222)** |
| --- | --- |
| Microbiological efficacy, *n* (%), 95% CI | 166 (79.0), 72.9-84.3 |
| Eradication | 124 (59.1) |
| Partial eradication | 13 (6.2) |
| Presumed eradication | 29 (13.8) |
| Presumed persistence | 5 (2.4) |
| Persistence | 39 (18.6) |
| Not evaluated | 6 |
| Overall clinical efficacy, *n* (%), 95% CI | 165 (78.2), 72.0-83.6 |
| Resolution | 39 (18.5) |
| Improvement | 126 (59.7) |
| Failure | 46 (21.8) |
| Not evaluated | 5 |
| Bacterial clearance, *n* (%), 95% CI |  |
| Yes | 150 (72.8), 66.2-78.8 |
| No | 56 (27.2) |
| Not evaluated | 10 |
| Duration of mechanical ventilation, days |  |
| Mean ± SD | 16.7 ± 15.9 |
| Median (IQR) | 14.0 (9.0, 20.0) |
| Min, Max | 0, 123 |
| ICU length of stay, days |  |
| Mean ± SD | 24.6 ± 14.8 |
| Median (IQR) | 21.0 (15.0, 28.0) |
| Min, Max | 0, 112 |
| Total hospital length of stay, days |  |
| Mean ± SD | 31.5 ± 19.7 |
| Median (IQR) | 26.0 (19.0, 37.0) |
| Min, Max | 7, 123 |

IQR interquartile range, ICU intensive care unit
