# Supplementary material for: The efficacy and safety of colistimethate sodium in the treatment of carbapenem-resistant Gram-negative bacilli: a real-world observational study
Source: Front Cell Infect Microbiol. 2026 May 29;16:1742142. doi: 10.3389/fcimb.2026.1742142 (PMC13259746; doi:10.3389/fcimb.2026.1742142)
Supplement: Supplementary file 4 [file Table4.docx]

**Supplementary Table S4** The 30-day all-cause mortality and primary causes of death

| **30-day all-cause mortality** | **Values (*n*=222)** |
| --- | --- |
| Mortality, *n* (%) | 30 (19.8) |
| Causes of death, *n* (%) |  |
| Patient/family declined further therapy | 9 (30.0) |
| Treatment failure of index infection | 15 (50.0) |
| Underlying/comorbid illness progression | 5 (16.7) |
| Other (intestinal ulcer with bleeding, disease deterioration) | 1 (3.3) |

Note: A total of 44 deaths were recorded; six were excluded because the recorded date of death was erroneous, and another eight occurred >30 days after the first CMS dose. Consequently, 30 deaths were retained for the 30-day all-cause mortality analysis.
