# Supplementary material for: The efficacy and safety of colistimethate sodium in the treatment of carbapenem-resistant Gram-negative bacilli: a real-world observational study
Source: Front Cell Infect Microbiol. 2026 May 29;16:1742142. doi: 10.3389/fcimb.2026.1742142 (PMC13259746; doi:10.3389/fcimb.2026.1742142)
Supplement: Supplementary file 5 [file Table5.docx]

**Supplementary Table S5** The summary of adverse events (AE)

| **AE** | **Value, *n* (%) (*n*=222)** |
| --- | --- |
| Any AE | 6 (2.7) |
| Hepatic impairment^*^ | 1 (0.5) |
| Renal impairment^‡^ | 5 (2.3) |
| Grade ≥3 AE^†^ | 0 |
| Serious AE | 0 |
| AE leading to treatment interruption | 2 (0.9) |
| Hepatic impairment | 1 (0.5) |
| Renal impairment | 1 (0.5) |
| AE leading to death | 0 |
| Life-threatening AE | 0 |
| AE requiring or prolonging hospitalization | 0 |
| AE causing significant disability/incapacity | 0 |
| Congenital anomaly or birth defect | 0 |

* Hepatic impairment: grade unknown.

‡ Renal impairment: Grade 1 (n=1), Grade 2 (n=4).

† Graded per Common Terminology Criteria for Adverse Events (CTCAE) v5.0.
