# Supplementary material for: The efficacy and safety of colistimethate sodium in the treatment of carbapenem-resistant Gram-negative bacilli: a real-world observational study
Source: Front Cell Infect Microbiol. 2026 May 29;16:1742142. doi: 10.3389/fcimb.2026.1742142 (PMC13259746; doi:10.3389/fcimb.2026.1742142)
Supplement: Supplementary file 6 [file Table6.docx]

| **Subgroup** | **Any AKI, *n* (%)** | **AKI stage, *n* (column %) within subgroup** | | |
| --- | --- | --- | --- | --- |
|  |  | **Stage 1** | **Stage 2** | **Stage 3** |
| Overall | 45 (20.4) | 19 (42.2) | 14 (31.1) | 12 (26.7) |
| Normal baseline SCr (*n*=57) | 15 (26.3) | 6 (40.0) | 7(46.7) | 2 (13.3) |
| Low baseline SCr (*n*=116) | 10 (8.6) | 3 (30.0) | 2 (20.0) | 5 (50.0) |
| High baseline SCr (*n*=38) | 20 (52.6) | 10 (50.0) | 5 (25.0) | 5 (25.0) |
| AKI prolonging hospitalisation | 17 (7.7) | 5 (29.4) | 5 (29.4) | 7 (41.2) |
| Prior CKD (*n*=14) | 6 (42.9) | 1 (16.7) | 1 (16.7) | 4 (66.7) |

**Supplementary Table S6** The acute kidney injury (AKI) events by baseline creatinine category and prior chronic kidney disease (CKD)
